# Supplementary material for: Exploring novel genetic and hematological predictors of response to neoadjuvant chemoradiotherapy in locally advanced rectal cancer
Source: Front Genet. 2023 Aug 31;14:1245594. doi: 10.3389/fgene.2023.1245594 (PMC10501402; doi:10.3389/fgene.2023.1245594)
Supplement: Supplementary file 1 [file Table1.docx]

**Supplementary Table 1. Oligonucleotides primer sequences for qRT-PCR designed using NCBI Primer Blast.**

| **Gene Name (Ref Seq)** | **Sequence (5'->3')** |
| --- | --- |
| ***IDO1 (NM_002164.6)*** |  |
| Forward primer | GGGAAGCTTATGACGCCTGT |
| Reverse primer | CTGGCTTGCAGGAATCAGGA |
| ***CXCL9 (NM_002416.3)*** |  |
| Forward primer | TGAGAAAGGGTCGCTGTTCC |
| Reverse primer | GGGCTTGGGGCAAATTGTTT |
| ***CYBB (NM_000397.4)*** |  |
| Forward primer | TGTCAAGTGCCCAAAGGTGT |
| Reverse primer | CCCAACGATGCGGATATGGA |
| ***IL6 (NM_000600.5)*** |  |
| Forward primer | CCACCGGGAACGAAAGAGAA |
| Reverse primer | GAGAAGGCAACTGGACCGAA |
| ***GAPDH (NM_002046.5)*** |  |
| Forward primer | GACAGTCAGCCGCATCTTCT |
| Reverse primer | GCGCCCAATACGACCAAATC |
