# Supplementary figures and images for: Exploring novel genetic and hematological predictors of response to neoadjuvant chemoradiotherapy in locally advanced rectal cancer
Source: Front Genet. 2023 Aug 31;14:1245594. doi: 10.3389/fgene.2023.1245594 (PMC10501402; doi:10.3389/fgene.2023.1245594)

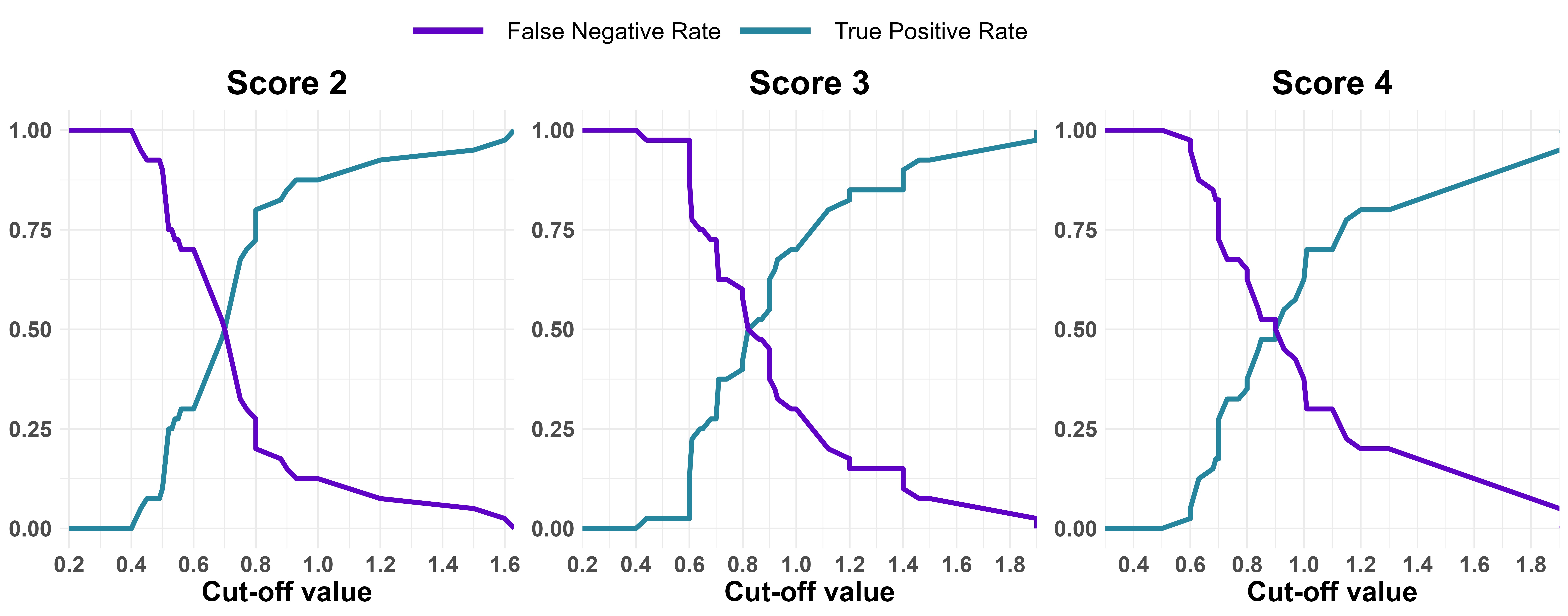

Supplement: Supplementary file 2 [file Image1.JPEG]
